# Supplementary material for: Lactobacillus casei Improve Anti-Tuberculosis Drugs-Induced Intestinal Adverse Reactions in Rat by Modulating Gut Microbiota and Short-Chain Fatty Acids
Source: Nutrients. 2022 Apr 17;14(8):1668. doi: 10.3390/nu14081668 (PMC9032531; doi:10.3390/nu14081668)
Supplement: Supplementary file 1 [file nutrients-14-01668-s001.zip › nutrients-1672095-supplementary.pdf]

Table S1. Nutrient composition of maintenance feed for experimental rats (g/kg).

**Moisture**	**≤100**	**Calcium**	**10–18**
Crude protein	≥180	total phosphorus	6–12
Crude fat	≥40	Calcium/total phosphorus	1.2:1–1.7:1
Crude fiber	≤50	Lysine	≥8.2
Crude ash	≤80	Methionine + cystine	≥5.3

The main raw materials are composed of: (1) Protein source: American chicken meal, Peruvian fish meal, soybean meal; (2) Fat source: vegetable oil; (3) Fiber source: bran; (4) Carbohydrate: corn, flour; (5) Vitamins: vitamins A, D, E, B and other complex vitamins; (6) Minerals: complex trace elements, calcium hydrogen phosphate, stone powder.

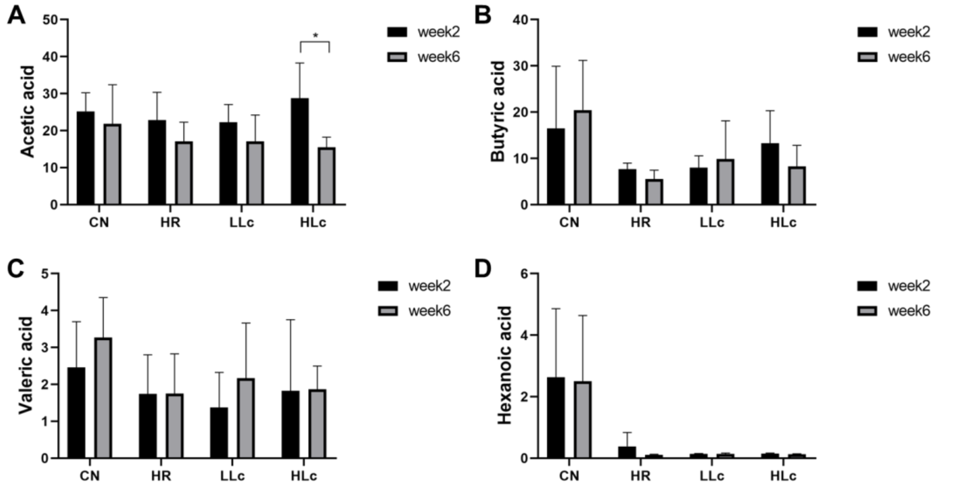

Figure S1. Comparison of short-chain fatty acid content between week 2 and week 6. (**A**), acetic acid; (**B**), butyric acid; (**C**), valeric acid; (**D**), hexanoic acid. * indicates $p < 0.05$. N = 4 in week 2 and N = 9 in week 6. CN: control group, HR: isoniazid + rifampicin model group, LLc: HR + low dose *L. casei ATCC334* group, HLc: HR + high dose *L. casei ATCC334* group.
